# Antidiabetic Potential and Antioxidant Activity of *Olea europaea* subsp. *Cuspidata* (Indian Olive) Seed Extracts

**DOI:** 10.1155/2022/5164985

**Published:** 2022-09-30

**Authors:** Muhammad Furqan Akhtar, Komal Maria Ashraf, Ammara Saleem, Ali Sharif, Hafiz Muhammad Zubair, Fareeha Anwar

**Affiliations:** ^1^Riphah Institute of Pharmaceutical Sciences, Riphah International University, Lahore Campus, Islamabad, Pakistan; ^2^Department of Pharmacy, The University of Lahore, Lahore, Pakistan; ^3^Department of Pharmacology, Faculty of Pharmaceutical Sciences, Government College University Faisalabad, Faisalabad, Pakistan; ^4^Institute of Pharmacy, Faculty of Pharmaceutical and Allied Health Sciences, Lahore College for Women University, Lahore, Pakistan

## Abstract

The aim of the present study was to evaluate the antioxidant and antidiabetic potential of Indian olive seed extracts. Plant seeds were sequentially extracted with n-hexane, chloroform, methanol, and water. 2,2-Diphenyl-1-picrylhydrazyl (DPPH) scavenging and alpha-amylase inhibitory activities of extracts were carried out. *Olea europaea* methanolic extract (MEOE) and aqueous extract (AEOE) were orally administered to normoglycemic and alloxan-treated diabetic rats so as to determine their hypoglycemic effect. High-performance liquid chromatography (HPLC) analysis showed gallic acid, ferulic acid, quercetin, and vanillic acid in MEOE. It was found that the methanolic and aqueous extracts exhibited the maximum DPPH and alpha-amylase inhibition activities, respectively. MEOE and AEOE exerted a significant decline in the fasting blood sugar in diabetic animals (*p* < 0.05); however, they did not cause hypoglycemia in nondiabetic animals. Treatment with MEOE and AEOE reduced the aggravated liver and kidney function biomarkers. Aggravated levels of oxidative stress biomarkers including superoxide dismutase (SOD), catalase (CAT), reduced glutathione (GSH), and malondialdehyde (MDA) were restored by treatment with MEOE. Moreover, MEOE improved the count of islets of Langerhans in the pancreas, fatty changes, and enlarged sinusoidal spaces in the liver and necrosis of the glomerulus and tubular cells of the kidney in diabetic rats. This study showed that the African olive seed extract effectively managed experimental diabetes and restored the normal functions and histology of the liver and kidney in diabetic rats through the reduction of oxidative stress.

## 1. Introduction

Diabetes mellitus (DM) is associated with an altered metabolism of lipids, proteins, and carbohydrates. It is the most widespread metabolic disorder in the world that affects about 2.8% population globally [1]. Antidiabetic agents such as biguanides and sulfonylureas are associated with severe adverse drug reactions such as weight gain, gastrointestinal disturbances, and liver and kidney damage. In the present era, there is a growing interest in herbal therapy owing to its safety, cost-effectiveness, and convenient availability. Several herbs are traditionally known as a remedy for DM. The whole plant or a part of it is used to treat serious disorders such as DM. Chronic hyperglycemia is often accompanied by oxidative stress which results in serious complications and disorders. Oxidative stress can be reversed by the use of antioxidants which reduce oxidative damage and, thus, prevent the long-term complications of DM [2].

Several plants and their individual constituents have been investigated for antidiabetic potential in animal and human studies. More than five hundred plants belonging to 140 different genera have exhibited antidiabetic activity when investigated on the basis of ethnomedicinal significance [3]. Extracts prepared from *Olea europaea* leaves showed in vitro antidiabetic and antioxidant activities [4]. Moreover, various phenolic acids, oleanolic acid, and oleuropein isolated from Olea europaea leaves have been attributed to the in vitro antidiabetic and antioxidant activities [5]. A previous study of olive leaves on high fat-fed rats treated with a low dose of streptozotocin demonstrated a reduction of body weight and control of hyperglycemia [6].

*Olea europaea* subsp. *Cuspidata* (Wall. and G. Don) Cif. belongs to the family Oleaceae and is commonly called as wild olive, African olive, Kao, and Kahu [7]. It is traditionally used for the treatment of toothache, rheumatism, backache, burn, diabetes, and asthma. It is also used as an astringent, antiseptic, diuretic, and rubefacient [8]. Maslinic acid and phenolic compounds isolated from *Olea europaea* have exhibited antioxidant activity [9]. Various studies have demonstrated the promising antihypertensive, antibacterial, and antinociceptive potential of *Olea europaea* [10].

This study was carried out to evaluate the antioxidant, *in vitro,* and *in vivo* antidiabetic potential of different extracts of *Olea europaea* subsp*. Cuspidata* seeds so as to demonstrate their effectiveness in combating DM.

## 2. Materials and Methods

Chloroform, n-hexane, dimethyl sulfoxide (DMSO), and sodium hydroxide were acquired from RDH®, Germany. Vitamin C, 3,5-dinitro-salicylic (DNS) acid, 2,2-diphenyl-1-picrylhydrazyl (DPPH), alpha-amylase, and starch were obtained from Unichem®, UK. Sodium potassium tartrate and alloxan monohydrate (Applichem®, Germany), glimepiride (Prudence Pharma Chem®, India), and Glucometer (Roche Inc.) were also used in the study.

### 2.1. Collection and Extraction of Plant

Seeds of *Olea europaea* subsp. *Cuspidata* were collected from the subtropical region of Pakistan (31°25′3.05″N, 73°04′23.56″E). The plant was identified by the taxonomist at GC University, Lahore, who assigned voucher no LAH^#^322017B. Seeds were washed, dried, and ground to coarse form. For extraction, 200 gm of ground material was extracted with 1 L of n-hexane. The coarse powder was then extracted with equal volumes of chloroform, methanol, and distilled water sequentially by the Soxhlet apparatus. The extracts were collected and dried at 40°C with a rotary evaporator. Semisolid extracts were stored in a refrigerator until further evaluation [11].

### 2.2. Phytochemical Analysis

Qualitative phytochemical screening of different plant extracts was carried out according to the previously described standard procedures [11].

### 2.3. High-Performance Liquid Chromatography (HPLC) Analysis

The chemical analysis of the methanolic extract of *Olea europaea* (MEOE) was carried out to quantify the phenolic acids and flavonoids by following the previous method. The plant extract (25 mg) was dissolved in 8 ml methanol and 12 ml water to prepare a sample for analysis. The mobile phase is comprised of two solvents with gradient elution. Solvent A consisted of methanol and acetonitrile at a ratio of 30 : 70, while solvent B comprised of water and acetic acid at a ratio of 6 : 94. A 20 *μ*l sample was injected to reverse phase HPLC (Perkin Elmer®, USA) equipped with a C18 column. The analytes were identified by matching with retention times of standard phytochemicals with a UV spectrum obtained at 275 nm, and the quantities were estimated from the area of standard phytochemicals [11].

### 2.4. In Vitro Antioxidant Activity

The free radical scavenging activity of all extracts was evaluated by the DPPH inhibition method [12]. A 40 mg DPPH was dissolved in 100 ml methanol to prepare 0.4 mM DPPH solution. A 2 ml DPPH solution admixed with 2 ml methanol served as a control solution. Various dilutions of each extract prepared with methanol were evaluated for antioxidant activity. 1 ml each of methanol and test solution were mixed with 2 ml DPPH solution in a test tube. All dilutions were prepared and tested in triplicates. Test tubes containing reacting solutions were wrapped in aluminum foil and allowed to stand in dark at 25°C. Absorbance was measured at 517 nm using a spectrophotometer 30 min after incubation. Ascorbic acid was used as a standard antioxidant.

### 2.5. In Vitro Alpha-Amylase Inhibitory Activity

For an appraisal of *in vitro* antidiabetic activity of different extracts, alpha-amylase inhibitory activity was carried out by following the procedure described earlier [12]. A 2.36 g DNS was dissolved in 80 ml 0.5 N NaOH solution by mixing on a sonicator for 30 min at 70°C. Then, 30 g sodium potassium tartrate was mixed with the previous solution. The final volume was made up to 100 ml with distilled water. The starch solution was prepared by dissolving 0.02 g starch in 20 ml of 20 mM sodium phosphate buffer (pH 6.9). The alpha-amylase solution was prepared by dissolving 0.0253 g of alpha-amylase in 100 ml distilled water.

A 1 ml alpha-amylase solution was added to an equal volume of the extract solution and incubated for 10 min at 25°C. Then, 1 ml starch solution was added to it. The reaction mixture was incubated again for 30 min at 37°C followed by the addition of 1 ml DNS reagent as a stop solution. The reaction mixture was finally incubated for 5 min in a boiling water bath, cooled to 25°C, and the final volume was made up to 10 ml by adding distilled water. Absorbance was determined at 540 nm with a UV-visible spectrophotometer and percentage inhibition was calculated. Control incubation, showing 100% enzymatic activity, was prepared in the same way. However, the extract was replaced with the solvent in the control solution. Acarbose was used as a standard alpha-amylase inhibitor.

### 2.6. Experimental Animals

Wistar rats of both sex (8–10 weeks old, weighing approximately 200–250 g) acquired from the University of Lahore were housed in stainless steel cages at 25 ± 02°C with a 12-hour day and night cycle with free access to standard pellet diet and water. The study was approved and conducted according to the guidelines of the Animal Ethics committee, the University of Lahore.

### 2.7. Hypoglycemic Activity in Nondiabetic Rats

Normoglycemic Wistar rats were orally given 250, 500, and 750 mg/kg MEOE and aqueous extract (AEOE) for one week. Fasting blood glucose (FBG) in rats was tabulated daily with a glucometer [13].

### 2.8. Alloxan-Induced Diabetes in Rats

A single intraperitoneal dose of alloxan monohydrate (150 mg/Kg) was administered to Wistar rats for the induction of DM [14]. The serum FBG of animals was measured three days after alloxan administration. Rats with FBG above 200 mg/dl were considered diabetic and used in the studies.

Group 1 was comprised of nondiabetic rats which were orally administered with normal saline. Group 2 served as a standard control in which diabetic rats were treated orally with a standard antidiabetic agent, Glimepiride (0.2 mg/kg). Group 3 was comprised of diabetic rats (negative control) which received normal saline only. Other groups comprised of diabetic rats receiving an oral daily dose of 250, 500, or 750 mg/Kg of aqueous or methanolic extract. Treatment was carried out for 14 consecutive days. Blood samples were collected from tail veins to determine FBG with the glucometer. On the 15^th^ day, the rats were anesthetized. Blood was collected by heart puncture for the determination of biochemical markers. Anesthetized rats were killed by cervical dislocation to remove the kidney and liver for histopathological evaluation. The complete blood count (CBC) and liver function tests (LFTs) and renal function tests (RFTs) were carried out on blood serum.

Isolated rat organs (liver and kidney) were kept in 10% formaldehyde. Small parts of the kidney and liver were embedded in paraffin wax, and tissue sections (5 *μ*m thickness) were prepared with a rotary microtome. The tissues embedded in paraffin were stained with eosin and hematoxylin [13]. Stained tissues were visualized under a compound microscope for pathological alterations.

### 2.9. Oxidative Stress Markers

For determining oxidative stress markers in the liver and kidney of rats, the 10% w/v tissue homogenates were prepared in phosphate buffer saline (PBS) pH 7.4 followed by centrifugation at 3000 rpm to collect the supernatant. The protein content in the tissues was estimated by the Lowry method. The oxidative stress biomarkers were determined by following earlier procedures [15].

### 2.10. Superoxide Dismutase (SOD) Activity

To determine the SOD activity in liver and kidney tissue homogenates, equal volumes of tissue homogenate and pyrogallol solution (0.1 ml) were mixed with 2.8 ml of PBS. Changes in absorbance were determined 5 min apart with a UV-Vis spectrophotometer at 325 nm. The activity of SOD was expressed as U/mg of protein.

### 2.11. Catalase (CAT) Activity

To determine the CAT activity, 50 *μ*l supernatant, 1.95 ml phosphate buffer of pH 7, and 1 ml 30 mM hydrogen peroxide were admixed, and absorbance was determined at 240 nm 0.5 min apart with UV-Vis spectrophotometer. The activity of CAT was expressed as U/mg of protein.

### 2.12. Reduced Glutathione (GSH) Level

To determine the GSH level in the liver and kidney tissue homogenates, 1 ml tissue supernatant was precipitated with an equal volume of 10% trichloroacetic acid solution followed by the addition of 4 ml PBS. The absorbance of the resultant solution was determined after adding 0.5 ml 5,5′-dithiobis-2-nitrobenzoic acid (DTNB) reagent. The GSH amount was expressed as *μ*g/mg of protein.

### 2.13. Malondialdehyde (MDA) Level

The level of MDA in the liver and kidney tissue homogenates was determined by mixing 1 ml tissue homogenate with 3 ml thiobarbituric acid reagent. The solution was thoroughly mixed and heated over a water bath. The absorbance of the cooled solution was determined at 532 nm which was obtained after centrifugation at 3000 rpm for 10 min. The amount of MDA was expressed as nM/ml.

### 2.14. Statistical Analysis

The data were presented as mean ± standard deviation. Statistical analysis of in vitro antioxidant and alpha-amylase inhibitory activities were performed by two-way analysis of variance (ANOVA) by GraphPad Prism®. The in vivo parameters were analyzed by one-way ANOVA. In case of statistically significant results, Tukey's post hoc evaluation was carried out.

## 3. Results

Qualitative phytochemical analysis revealed that the MEOE was comprised of carbohydrates, glycosides, saponin, proteins, fixed oils, tannins, and phenols. AEOE consisted of alkaloids, saponin, and proteins. Only saponins and fixed oils were present in chloroform and n-hexane extracts.

### 3.1. Chemical Characterization

HPLC analysis of the plant methanolic extract revealed the presence of gallic acid, ferulic, and quercetin. Vanillic acid (37.755 ppm) was present in the highest amount in the extract followed by ferulic acid, quercetin, and gallic acid. The phytochemicals detected in the plant extract along with the retention time, area, and concentration are presented in Table 1.

### 3.2. In Vitro Antioxidant Activity

In vitro free radical scavenging activity of seed extracts showed that the methanolic extract exhibited the maximum percentage inhibition (91.2%) at 500 *μ*g/ml concentration followed by chloroform (90.73%), aqueous (82.21%), and n-hexane (81.63%) at the same concentration.

**Figure 1:**
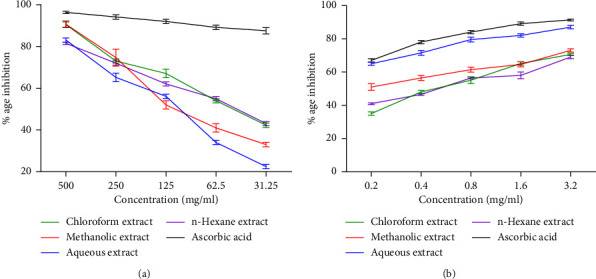
In vitro antioxidant and alpha-amylase inhibition activities of different extracts of *Olea europaea* subsp. *Cuspidata* seeds. (a) In-vitro antioxidant activity (b) in-vitro antidiabetic activity. Each value represents the mean with ±S.D. (*n* = 3).

The decreasing order of DPPH scavenging activity was methanolic > chloroform < aqueous >  n-hexane. IC50 of methanolic extract, aqueous extract, chloroform extract, n-Hexane extract, and ascorbic acid were 311.9, 118.1, 153.4, 112.5, and 136.3 *μ*g/ml, respectively. Detailed results of the free radical scavenging activity of all seed extracts are shown in Figure 1.

### 3.3. Alpha-Amylase Inhibitory Activity

It was also demonstrated that the aqueous extract exhibited the maximum percentage inhibition of alpha-amylase (82.10%) at 1.6 mg/ml concentration followed by chloroform (65.60%), methanol (64.90%), and n-hexane extract (58.25%). IC50 was of methanolic extract, aqueous extract, chloroform extract, n-Hexane extract, and acarbose of 1.331, 0.3194, 0.3730, 0.9374, and 0.1105 mg/ml, respectively. A concentration-dependent effect was observed as shown in Figure 1.

### 3.4. Effect on Nondiabetic Rats

**Figure 2:**
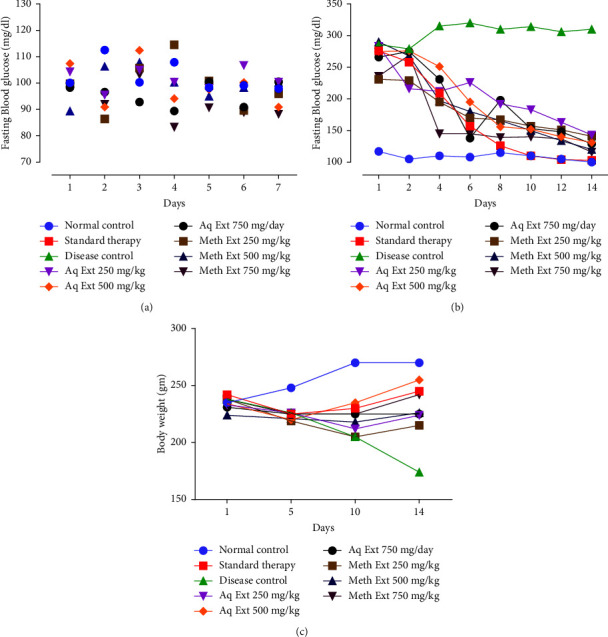
Effect of treatment with the methanolic and aqueous extracts of *Olea europaea* subsp. *Cuspidata* seeds on fasting blood glucose and body weight. (a) Blood glucose in Non-diabetic rats (b) Hypoglycemic effect in diabetic rats (c) Body weight of diabetic rats. Each value represents the mean ± S.D. (*n* = 6).

It was found that the statistically considerable difference was not observed in the FBG of normal rats and extract-treated rats at 250, 500, and 750 mg/kg/day dosage of the aqueous and methanolic extracts. The results of the effect of plant seed extracts on normoglycemic rats are shown in Figure 2.

### 3.5. Effect on Diabetic Animals

MEOE and AEOE (250, 500, and 750 mg/kg) in diabetic rats showed a remarkable hypoglycemic effect. It was revealed that all extract-treated groups showed a significant reduction FBG when compared to untreated diabetic rats. The FBG levels of untreated and treated diabetic animals are shown in Figure 2.

### 3.6. Body Weight of Diabetic Animals

It was seen that the administration of seed extracts had reversed weight loss in diabetic rats. A statistically significant difference in treated and untreated diabetic rats was found at day 14 (Figure 2).

### 3.7. Liver and Renal Function Markers

Damage to the liver and kidney damage was observed in diabetic animals from the abnormal values of alanine and aspartate aminotransferases, alkaline phosphatase, urea, and creatinine. There was a significant reduction (*p* < 0.05) in the level of renal and liver function markers of the extract-treated animals opposite to diseased animals (Table 2).

### 3.8. Blood Cell Count in Diabetic Rats

A statistically insignificant difference in blood cell count of the diabetic animals was observed in comparison to the normal rats. The plant extract did not induce significant changes in the blood cell count of white and red blood cells, hemoglobin, agranulocytes, granulocytes, platelets, hematocrit, and packed cell volume which were unaffected by extracts treatment.

### 3.9. Effect on SOD and CAT

**Figure 3:**
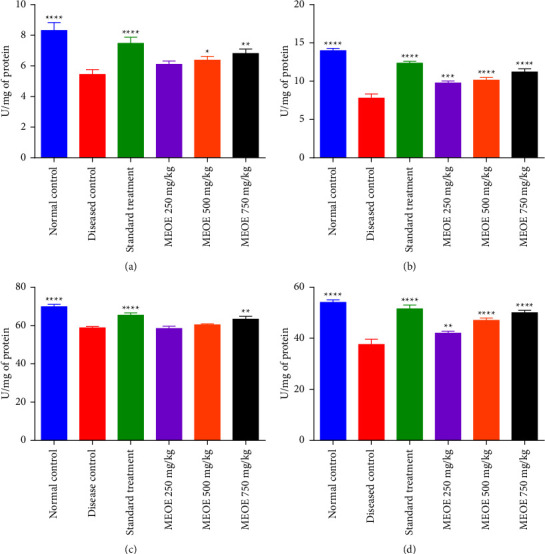
Effect of treatment with the methanolic extract of *Olea europaea* (MEOE) on superoxide dismutase (SOD) and catalase (CAT) in the liver and kidney of diabetic rats. (a) SOD in Liver (b) SOD in kidney (c) CAT in liver (d) CAT in kidney ^*∗*^,^*∗∗*^,^*∗∗∗*^, and ^*∗∗∗∗*^represent the significant difference in comparison with untreated diabetic rats at *P* < 0.05, 0.01, 0.001, and 0.0001, respectively.

The effect of MEOE on the SOD and CAT activities in the liver and kidney were significant. It was found that the diseased group showed significantly reduced activities of SOD and CAT in the liver and kidney homogenates as compared to normoglycemic rats. Administration of MEOE at 500 and 750 mg/kg significantly improved the SOD activity in the liver tissue homogenate of diabetic rats, while only 750 mg/kg dose of MEOE exhibited an increased activity of CAT in the liver of diabetic rats. Administration of MEOE at all dose levels increased the activities of SOD and CAT in the kidneys of diabetic rats in comparison to the diseased rats as shown in Figure 3.

### 3.10. Effect on GSH and MDA

**Figure 4:**
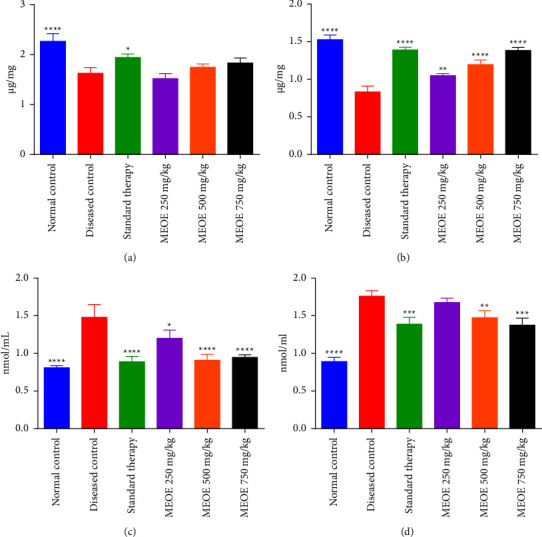
Effect of treatment with the methanolic extract of *Olea europaea* (MEOE) on reduced glutathione (GSH) and malondialdehyde (MDA) in the liver and kidney of diabetic rats. (a) GSH in liver (b) GSH in Kidney (c) MDA in liver (d) MDA in kidney ^*∗*^,^*∗∗*^,^*∗∗∗*^, and ^*∗∗∗∗*^represent the significant difference in comparison with untreated diabetic rats at *P* < 0.05, 0.01, 0.001, and 0.0001, respectively.

It was found that the administration of alloxan resulted in the reduction of GSH and an increase in MDA levels in the liver and kidney of diabetic rats. It was also revealed that the MEOE at all dose levels significantly increased the level of GSH in the kidneys of diabetic rats as compared to the disease control group. However, MEOE did not exhibit any significant improvement in the GSH level in the liver of diabetic rats. MEOE showed a statistically significant decrease of the MDA level in the liver of diabetic rats in comparison to the disease control group. Administration of MEOE at 250 mg/kg failed to reduce the MDA level in the kidney of diabetic rats in comparison to the disease control group; however, MEOE at 500 and 750 mg/kg caused a significant reduction in the MDA level of kidney in diabetic rats as shown in Figure 4. Glimepiride showed the most significant amelioration of GSH and MDA levels in diabetic rats as compared to the disease control group.

### 3.11. Histopathological Changes

**Figure 5:**
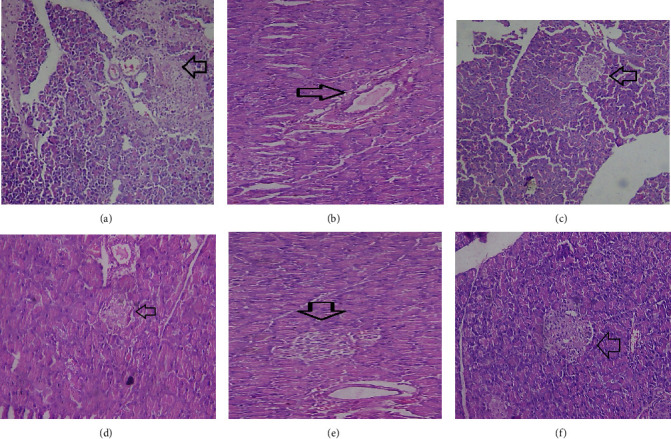
The effect of *Olea europaea* methanolic extract (MEOE) on the pancreas of the diabetic rats. (a) Normal control rats, (b) disease control group, (c) standard treatment, (d) MEOE 250 mg/kg, (e) MEOE 500 mg/kg, and (f) MEOE 750 mg/kg. Arrow shows the islet of Langerhans.

**Figure 6:**
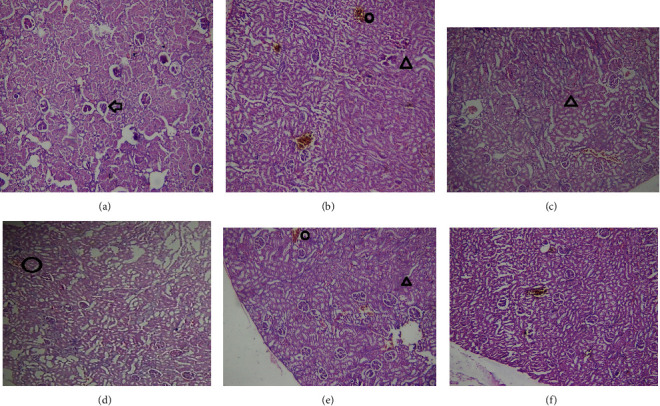
The effect of Olea europaea methanolic extract (MEOE) on the kidney of the diabetic rats. (a) Normal control rats, (b) disease control group, (c) standard treatment, (d) MEOE 250 mg/kg, (e) MEOE 500 mg/kg, and (f) MEOE 750 mg/kg. Arrow, circle, and triangle show glomerulus, thrombosis, and damaged tubules, respectively.

**Figure 7:**
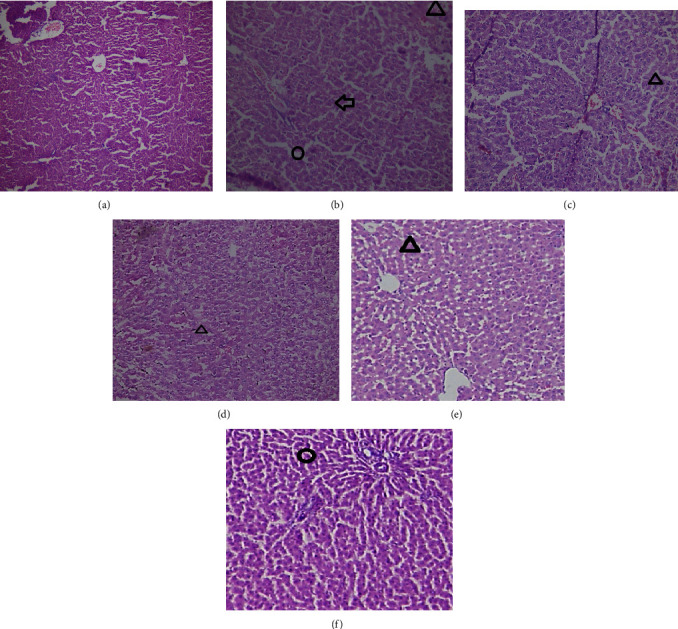
The effect of *Olea europaea* methanolic extract (MEOE) on the liver of diabetic rats. (a) Normal control rats, (b) disease control group, (c) standard treatment, (d) MEOE 250 mg/kg, (e) MEOE 500 mg/kg, and (f) MEOE 750 mg/kg. Arrow, triangle, and circle show fatty liver, necrosis, and sinusoidal spaces, respectively.

It was evident from the histological photomicrographs that the disease control rats had significantly damaged pancreas showing a reduced number of the islets of Langerhans. Administration of MEOE dose-dependently restored the islets of Langerhans as shown in Figure 5. Treatment with MEOE also decreased the thrombosis and inflammation of kidney blood vessels and alloxan-induced necrosis of the glomerulus and tubular cells. MEOE (750 mg/Kg) had the most prominent effect (Figure 6). Alloxan treatment also resulted in necrosis, fatty changes, and enlarged sinusoidal spaced in the liver of disease control rats. The histopathological evaluation of diabetic rats revealed that the treatment with MEOE ameliorated the fatty changes, steatohepatitis, and sinusoidal enlargement caused by alloxan treatment (Figure 7).

## 4. Discussion

The current study demonstrated the antioxidant and antidiabetic potential of *Olea europaea* seed extracts. It was found that plant extracts had shown a concentration-dependent increase in free radical scavenging and alpha-amylase inhibition activities. MEOE demonstrated an increase in the body weight and controlled FBG along with the improvement of LFTs and histological lesions in the pancreas, kidney, and liver in the diabetic rats. Administration of MEOE resulted in an improvement of CAT, SOD, and GSH, a reduction of MDA.

A current study on olive seeds showed that the MEOE had large contents of phenolic acids such as ferulic acid, gallic acid, and vanillic acid. A previous study showed that 37.75 mg/kg vanillic acid was present in the olive seed extract as compared to 0.67–4 mg/kg in olive oil [16]. Moreover, ferulic acid, gallic acid, and quercetin along with their derivatives were also detected and quantified in olive oil [17].

It was found that all seed extracts showed a varying level of DPPH and alpha-amylase inhibition activities (Figure 1). A previous study reported the alpha-amylase inhibitory activity of extracts obtained from the leaves of olive [18]. Our study showed that the different extracts of olive seeds demonstrated an IC50 for alpha-amylase inhibition between 0.11 and 1.33 mg/ml in comparison to an IC50 of 0.2 to 4 mg/ml exhibited by leaf extract of olive trees. Moreover, the DPPH scavenging activity of various extracts of olive seeds demonstrated an antioxidant effect in the current study similar to the different leaf extracts of olive trees [19]. This study confirmed that the seeds of Indian olive had exhibited dose-dependent DPPH and alpha-amylase inhibitory potential that might be attributed to the presence of natural phenols and flavonoids [20].

A statically nonsignificant hypoglycemic effect was seen in normoglycemic animals which showed that the olive seed extracts might be devoid of any hypoglycemic effect in normoglycemic individuals in contrast to the hypoglycemic effect in diabetic rats [21]. It is also well documented that the continuous weight loss occurred as the DM progressed. Treatment with either extract significantly increased (*p* < 0.05) the body weight of rats than untreated diabetic rats. It also showed that *Olea europaea* subsp. *Cuspidata* had tried to restore the body weight possibly through normalizing metabolic processes of the body. However, there was no significant difference in the hematological parameters of the experimental animals which showed that the administration of aqueous and methanolic extracts were devoid of any toxicity [22]. The liver and kidney function tests revealed that the administration of aqueous or methanolic extract improved the liver and kidney function of diabetic rats. The liver and renal functions were improved and accompanied by the reduction in alloxan-induced lesions in the liver and kidney. Seed extracts of some other plants, such as *Citrullus colocynthis*, had also shown hypoglycemic potential and body weight gain in diabetic rats [23]. It can be speculated that the antioxidant nature of olive seed extracts might have contributed in the improvement of liver and kidney function tests and histopathological changes [24].

It is previously established that alloxan administration had resulted in the generation of reactive oxygen species (ROS) through cyclic redox reactions of dialuric acid through metabolism [25]. Thus, dialuric acid acts as a free radical producer and damages the pancreas, liver, and kidney. This generation of free radicals leads to oxidative stress resulting in cytotoxic effects in different organs [26]. Damage in the liver in the form of fatty changes and steatohepatitis occurs. Previous research confirms the decrease in GSH, SOD, and CAT and an increased lipid peroxidation in the liver of diabetic animals [27]. Some phytochemicals present in the olive leaves such as oleuropein and hydroxytyrosol had shown not only in vitro and in vivo antioxidant properties but also exhibited antidiabetic potential through reduction of lipid peroxidation [12]. Improvement in the liver and renal function markers also depicts normalized metabolic function because of reduced generation of ROS which indirectly confirms the reduction of oxidative stress [28]. Quercetin in MEOE is found to exhibit a reduction in oxidative stress in alloxan-treated diabetic mice by preventing lipid peroxidation and increasing the SOD, CAT, and GSH [29]. Moreover, gallic acid, ferulic acid, and vanillic acid have demonstrated the antihyperglycemic effect in diabetic animal, and prevented diabetes-related liver and kidney damage through their antioxidant and anti-inflammatory roles [1, 2, 30].

This study shows that African olive seed extracts slightly restored body weight possibly by normalizing the metabolic processes of the body. Evaluation of complete blood count among untreated and treated diabetic rats showed that the treatment with MEOE was devoid of any pathological changes in blood cell count [2].

It is evident from the current study that MEMO reduced the alloxan-induced hepatic damage in diabetic rats. Previously, it is known that olive oil had reduced the hepatotoxicity induced by ethanol or acetaminophen by the reduction of oxidative stress due to the presence of phytochemicals such as gallic acid [31]. Moreover, MEMO reduced the alloxan-induced lesions in nephrons of diabetic rats. The nephroprotective effect of olive oil was demonstrated previously against gentamicin-induced nephrotoxicity through the reduction of oxidative stress.

## 5. Conclusion

In the light of the above findings, it can be concluded that *Olea europaea* subsp. *Cuspidata* seeds were helpful in managing DM due to their antioxidant and alpha-amylase inhibitory activities. Antioxidant and antidiabetic activities of the plant seed extract may be due to the presence of specific phytoconstituents such as gallic acid, quercetin, ferulic acid, and vanillic acid which resulted in a reduction of oxidative stress. The seeds of African olive may serve to relieve the long-term complications associated with DM. There is a need to elucidate the molecular mechanism involved to treat diabetes.

## Figures and Tables

**Table 1:** Phytochemicals detected by HPLC in Indian olive seed extract.

Sr. No.	Phytochemical	Retention time	Area	Concentration (ppm)
1	Gallic acid	2.899	403,527.0	14.728
2	Vanillic acid	8.037	858,081.7	37.755
3	Ferulic acid	12.904	684,013.6	33.858
4	Quercetin	25.001	1849,580.8	17.571

**Table 2:** Effect of methanolic and aqueous extracts of *Olea europaea* subsp. *Cuspidata* seeds on the liver and kidney function tests in diabetic rats.

Parameters	Normal control	Diabetic control	Standard therapy	Aqueous extract (mg/kg/day)			Methanolic extract (mg/kg/day)		
				250	500	750	250	500	750
Alanine aminotransferase (U/L)	49 ± 6.47	167 ± 6.15	55 ± 1.25^*∗*^	61 ± 3.35^*∗*^	50 ± 4.32^*∗*^	37 ± 2.10^*∗*^^#^	60 ± 5.3^*∗*^	53 ± 4.23^*∗*^	42 ± 5.36^*∗*^
Aspartate aminotransferase (U/L)	106 ± 4.16	165 ± 4.23	105 ± 6.02^*∗*^	141 ± 5.48^*∗*^	130 ± 6.54^*∗*^	104 ± 5.36^*∗*^	156 ± 5.61	136 ± 4.36^*∗*^	135 ± 1.47^*∗*^
Alkaline phosphatase (U/L)	142 ± 5.01	388 ± 5.02	251 ± 4.56^*∗*^	193 ± 4.18^*∗*^^#^	177 ± 5.02^*∗*^^#^	77 ± 4.35^*∗*^^#^	189 ± 8.66^*∗*^^#^	151 ± 6.78^*∗*^^#^	106 ± 1.62^*∗*^^#^
Bilirubin total (mg/dl)	0.1 ± 0.05	0.1 ± 0.02	0.1 ± 0.02	0.1 ± 0.03	0.2 ± 0.04	0.1 ± 0.02	0.08 ± 0.001	0.09 ± 0.001	0.1 ± 0.01
Bilirubin direct (mg/dl)	0.01 ± 0.001	0.01 ± 0.001	0.03 ± 0.01	0.02 ± 0.01	0.05 ± 0.01	0.02 ± 0.001	0.2 ± 0.02	0.2 ± 0.01	0.01 ± 0.001
Total protein (g/dl)	6.7 ± 1.24	5.2 ± 0.32	6.1 ± 1.02	6.2 ± 0.22	6.4 ± 0.14	6.8 ± 0.05	5.6 ± 0.44	4.9 ± 0.61	7 ± 0.05
Albumin (g/dl)	4.5 ± 1.01	3.3 ± 0.21	4.6 ± 0.14	4.4 ± 0.01	4.8 ± 0.03	4.8 ± 0.01	4.6 ± 0.12	4.9 ± 0.21	5 ± 0.01
Gama glutamyl transferase (U/L)	0.1 ± 0.04	37 ± 4.21	7 ± 1.54	16 ± 1.26	6.2 ± 0.12	6.2 ± 0.35	6.2 ± 0.02	4.2 ± 0.03	4.1 ± 0.07
Urea (mg/dl)	58 ± 5.61	460 ± 6.58	36 ± 4.25^*∗*^	68 ± 3.60^*∗*^^#^	57 ± 4.56^*∗*^^#^	46 ± 1.54^*∗*^	64 ± 3.32^*∗*^^#^	54 ± 2.13^*∗*^^#^	48 ± 5.69^*∗*^
Creatinine (mg/dl)	0.4 ± 0.07	5.9 ± 0.32	0.3 ± 0.05^*∗*^	0.3 ± 0.05^*∗*^	0.3 ± 0.04^*∗*^	0.3 ± 0.01^*∗*^	0.9 ± 0.02^*∗*^	0.4 ± 0.05^*∗*^	0.4 ± 0.01^*∗*^

Values were presented as mean with +S.D (*n* = 6). ^*∗*^represents the significant difference with untreated diabetic rats and ^#^shows statistical difference with standard therapy at *P* < 0.05.

## Data Availability

The data used to support the findings of this study are included in the article.
